# Legislation

**Published:** 1884-06

**Authors:** 


### LEGISLATION.

The villainous abuses of our legislative system came very near a practical illustration during the closing hours of the session of the Legislature of the State of New York. It is notorious that during the hurry and heedlessness of the last days, when nearly every member has some pet scheme to consummate, advantage is taken to push through, without examination, all sorts of questionable bills. The title often gives no idea of the contents, and the whole bill is so drawn as to mislead and deceive those who do read it. Every means is taken to distract attention from it, in the hope that it will get through without challenging enquiry. Such a bill, one that would have quite destroyed the usefulness of the statute regulating the practice of dentistry in the State of New York, and have made it entirely inoperative, was within a hair's breadth of its passage during the last week of the session of the Legislature. The mere accident of a member of the Dental Society of the State of New York, who knows the devious tricks of legislators, happening to drop into the Assembly Chamber when the bill came up for its third reading, alone prevented its being a law to-day. It had already passed the Senate, and another half-hour would have seen it safely through the Assembly. There was some lively work done to head it off, but fortunately the matter was taken in hand by ex-State Senator Dr. A. M. Holmes, and its reference back to the committee was secured, where it was effectually squelched. But the admirers of our excellent law will in future understand that there are various classes of men who will bear watching, and they will do well to keep their eyes open during the sessions of coming Legislatures. Heedless or indifferent to its effects upon our own citizens, at the mere request of carpet-baggers from other States who were disqualified for legal practice, a legislator who must hold very peculiar views as to his duties to his constituents attempted to make New York a general refuge for quacks by introducing the following bill, which we commend to the especial notice of the profession in Brooklyn:

SENATE BILL NO. 360, INTRODUCED BY MR. DAGGETT.

AN ACT for the relief of certain persons engaged in the regular practice of dentistry.

*The People of the State of New York, represented in Senate and Assembly, do enact as follows:*

SECTION 1. Any person who was engaged in the regular practice of dentistry within any state of the United States except this State, on the twentieth day of June, eighteen hundred and seventy-nine, and who would have been entitled to registration as a dentist, as provided in the third section of chapter five hundred and forty of the laws of eighteen hundred and seventy-nine, entitled "An act to regulate the practice of dentistry in the state of New York," had he been so practicing within this state, and who shall make and file with the clerk of the county in which he registers his affidavit to the effect that he was so engaged in such practice of dentistry, may, within sixty days after the passage of this act, cause his name, office and post-office address to be registered in the county clerk's office in the manner provided in said third section of said act, and such registration shall have like force and effect as if made within the time prescribed by said section of said act, according to its provisions. Any person who shall willfully make and file a false affidavit for the purpose of procuring such registration shall be subject to conviction and punishment for perjury.

SEC. 2. This act shall take effect immediately.

